# Change in Body Fat Mass Is Independently Associated with Executive Functions in Older Women: A Secondary Analysis of a 12-Month Randomized Controlled Trial

**DOI:** 10.1371/journal.pone.0052831

**Published:** 2013-01-07

**Authors:** Elizabeth Dao, Jennifer C. Davis, Devika Sharma, Alison Chan, Lindsay S. Nagamatsu, Teresa Liu-Ambrose

**Affiliations:** 1 Department of Physical Therapy, University of British Columbia, Vancouver, British Columbia, Canada; 2 Collaboration for Outcomes Research and Evaluation, Vancouver, British Columbia, Canada; 3 Department of Psychology, University of British Columbia, Vancouver, British Columbia, Canada; 4 Brain Research Centre, University of British Columbia, Vancouver, British Columbia, Canada; Pennington Biomed Research Center, United States of America

## Abstract

**Objectives:**

To investigate the independent contribution of change in sub-total body fat and lean mass to cognitive performance, specifically the executive processes of selective attention and conflict resolution, in community-dwelling older women.

**Methods:**

This secondary analysis included 114 women aged 65 to 75 years old. Participants were randomly allocated to once-weekly resistance training, twice-weekly resistance training, or twice-weekly balance and tone training. The primary outcome measure was the executive processes of selective attention and conflict resolution as assessed by the Stroop Test. Sub-total body fat and lean mass were measured by dual-energy x-ray absorptiometry (DXA) to determine the independent association of change in both sub-total body fat and sub-total body lean mass with Stroop Test performance at trial completion.

**Results:**

A multiple linear regression model showed reductions in sub-total body fat mass to be independently associated with better performance on the Stroop Test at trial completion after accounting for baseline Stroop performance, age, baseline global cognitive state, baseline number of comorbidities, baseline depression, and experimental group. The total variance explained was 39.5%; change in sub-total body fat mass explained 3.9% of the variance. Change in sub-total body lean mass was not independently associated with Stroop Test performance (***P***>0.05).

**Conclusion:**

Our findings suggest that reductions in sub-total body fat mass – not sub-total lean mass – is associated with better performance of selective attention and conflict resolution.

## Introduction

As the world's population ages, dementia will become a global epidemic [1]. Currently it is estimated that 5.2 million people have Alzheimer's disease (AD) in the United States (US) alone [1]. The economic burden of dementia in the US will rise dramatically from $200 billion in 2012 to $1.1 trillion in 2050 [1]. Thus, it is a priority to identify effective prevention strategies that will combat the increasing burden dementia imposes on our population. Current evidence demonstrates physical activity is an effective prevention strategy for cognitive decline [2], [3], [4], [5], [6].

Physical activity has been identified as an effective intervention for maintaining and improving cognitive performance through promoting brain health in older adults. Directly, exercise increases brain availability of several classes of growth factors, most importantly brain-derived neurotrophic factor [7]. Brain-derived neurotrophic factor enhances synaptic transmission, encodes long term potentiation, improves learning, promotes differentiation, neurite extension, and protects against ischemic insults and thus plays a crucial role in neuroplastic, neurotrophic, and neuroprotective factors [7]. Brain-derived neurotropic factor also supports the health and functioning of glutamatergic neurons in the hippocampus, a brain region important in learning and memory and is the site of early deterioration in neurodegenerative diseases like AD [8].

Indirectly, exercise may promote brain health by reducing vascular risk factors such as hypertension [9], cardiovascular [10], and cerebrovascular disease [11]. As these chronic conditions are highly associated with increased body mass index (BMI) [12], regular physical activity may also promote cognitive function in older adults by reducing adipose tissue. Increased adiposity can cause carotid-artery-wall thickening, arterial stiffness, and vascular and coronary endothelial dysfunction contributing to vascular diseases [13]. For example, these mechanisms have been linked to brain pathologies associated with vascular dementia [14], such as lacunar infarcts and white matter lesions [15]. Adipose tissue also secretes various bioactive metabolites (i.e., transforming growth factor β [16], tumour necrosis factor α [17], angiotensin II [18], cytokines [19], fatty acids [20]) that have been associated with dementia.

Although adiposity has been linked to reduced brain health [13], the relationship between adiposity and cognitive function remains equivocal. Findings from cross sectional [21], [22] and prospective cohort [23], [24], [25] studies report both positive and negative cognitive outcomes with increased adiposity. For example, waist circumference, waist-hip-ratio, and visceral adiposity were inversely related to cognitive function in both older men and women [21], [22]. Another study implicated decreased central obesity as a key factor in cognitive decline in older women after adjusting for potential confounding factors for cognitive function (i.e., age, sex, level of education, and depression) and health conditions (i.e., hypertension, diabetes, and smoking status) [23]. Further, increased adiposity over time was associated with positive change in cognitive function in older men when obese at baseline [23]. Conversely, in the Health, Aging and Body Composition (ABC) Study [24], higher levels of subcutaneous fat and total fat mass were associated with worsening global cognitive function in men after controlling for metabolic disorders, adipocytokines, and sex hormone levels. No association between adiposity and cognitive change was found in older women in both the Health ABC Study [24] and the Women's Health Initiative Study of Cognitive Aging [25].

Furthermore, the association between adiposity and incident dementia remain unclear [26], [27], [28], [29]. Obesity in mid-life appears to increase the risk for cognitive decline and dementia in late-life [28], [29]. This association is reversed in adults over 65 years of age; higher BMI in late life is associated with a reduced risk of dementia [26], [27]. Research suggests that low BMI in late life may be an early pathological sign of dementia [26], [27].

Several factors may contribute to the discrepant findings in the adiposity and cognitive function literature. First, increased age is often characterized by a loss in lean body mass and an increase in adipose tissue [30]. Thus, BMI is an insensitive measure of body composition in older adults as it does not reflect this change in body composition [31]. Second, many of the past studies were cross sectional hence no temporal associations were established and unknown and known confounders were not controlled for [21], [32], [33]. Third, previous studies have relied on measures of global cognitive function such as the Mini-Mental State Examination (MMSE) [23], [24] which is not sensitive to subtle changes in cognitive function in healthy older adults [34]. Lastly, to our knowledge only one study to date has assessed the effect of *change* in body fat mass on cognitive performance in healthy community-dwelling older adults [23] and no study has addressed the effect of *change* in body lean mass. Yet, such knowledge would facilitate the development and refinement of targeted interventions to improve cognitive function in older adults. For example, if reduced body fat mass – rather than increased body lean mass – was independently associated with improved cognitive performance, it would justify the promotion of targeted exercise training interventions that reduce fat mass (i.e., aerobic training) rather than those that increase lean mass (i.e., progressive resistance training).

Further, few studies have specifically assessed the effect of adipose tissue on executive functions. Executive functions are higher-order cognitive processes that controls and manages other cognitive abilities. It allows for effective goal-directed behaviour and control of attentional resources which are necessary for managing everyday activities and functional independence [35]. Normal aging is associated with a decrease in cognitive resources responsible for executive functions, in particular the capacity to execute tasks that involve selective attention and conflict resolution [36]. These cognitive domains as measured by the Stroop Test [37] has been significantly associated with impaired mobility [38] and instrumental activities of daily living [39]. Executive functions are also highly relevant to healthy aging as it is a predictor of conversion to AD [40].

Thus, we conducted a secondary analysis on data collected from a 12-month randomized controlled trial of exercise to investigate the independent association of change in both sub-total body fat mass and sub-total body lean mass with executive functions, specifically the executive processes of selective attention and conflict resolution, at trial completion.

## Methods

### Ethics Statement

Ethical approval was obtained from the Vancouver Coastal Health Research Institute (V06-0326) and the University of British Columbia's Clinical Research Ethics Board (H06-0326). All participants provided written informed consent.

### Study Design and Participants

The sample for this secondary analysis consisted of a subset of 155 women who consented and completed a 12-month randomized controlled trial of exercise that primarily aimed to examine the effect of once-weekly or twice-weekly resistance training compared with a twice-weekly balance and tone exercise intervention on executive functions [41]. The design and the primary results of the study have been previously reported. Of the 155 women recruited, 114 women underwent a DXA scan and were included in this secondary analysis.

We recruited and randomized senior women who: 1) were aged 65–75 years; 2) were living independently in their own home; 3) obtained a score ≥24 on the MMSE [42]; and 4) had a visual acuity of at least 20/40, with or without corrective lenses. We excluded those who: 1) had a diagnosed neurodegenerative disease (e.g., AD) and/or stroke; 2) were taking psychotropic drugs; 3) did not speak and understand English; 4) had moderate to significant impairment with ADLs as determined by interview; 5) were taking cholinesterase inhibitors within the last 12 months; 6) were taking anti-depressants within the last six months; or 7) were on oestrogen replacement therapy within the last 12 months.

### Randomization

The randomization sequence was generated by www.randomization.com and was concealed until interventions were assigned. This sequence was held independently and remotely by the Research Coordinator. Participants were enrolled and randomised by the Research Coordinator to one of three groups: once-weekly resistance training (n = 37), twice-weekly resistance training (n = 41), or twice-weekly balance and tone (n = 36).

### Exercise Intervention

#### Resistance Training

All classes were 60 minutes in duration. The protocol for this program was progressive and high-intensity in nature. Both a Keiser® Pressurized Air system and free weights were used to provide the training stimulus. Other key strength exercises included mini-squats, mini-lunges, and lunge walks.

#### Balance and Tone

This program consisted of stretching exercises, range of motion exercises, kegals, balance exercises, and relaxation techniques. This group served to control for confounding variables such as physical training received by traveling to the training centres, social interaction, and lifestyle changes secondary to study participation.

### Descriptive Variables

Age was measured in years. We used the 15-item Geriatric Depression Scale (GDS) [43] to screen for depression. Global cognition was assessed using the MMSE [42]. Functional Comorbidity Index (FCI) was calculated to estimate the degree of comorbidity associated with physical functioning [44]. This scale's score is the total number of comorbidities.

### Dependent Variable: Executive Processes of Selective Attention and Conflict Resolution

Our primary outcome measure was the executive cognitive processes of selective attention and conflict resolution, as measured by the Stroop Test. For the Stroop Test, we used three conditions. First, participants were instructed to read out words printed in black ink (e.g., blue). Second, they were instructed to read out the color of colored x's. Finally, they were shown a page with color words printed in incongruent colored inks (e.g., the word blue printed in red ink). Participants were asked to name the ink color in which the words are printed (while ignoring the word itself). There were 80 trials for each condition and we recorded the time participants took to read each condition. The ability to selectively attend and control response output was calculated as the time difference between the third condition and the second condition (i.e., interference score). Smaller time differences indicate better selective attention and conflict resolution.

### Independent Variables of Interest: Sub-total Body Fat Mass and Sub-Total Body Lean Mass

Sub-total body fat mass and sub-total body lean mass, which does not include the head/skull, were measured using DXA. This method uses a three-compartment model of body composition and provides an estimate of fat and lean mass. A whole body scan takes approximately six minutes and the total radiation exposure per session is less than 10 millirems, which is similar to the background radiation one would be exposed to during a one-way flight from Vancouver to Halifax on a commercial airline. The participants were instructed to lay supine on a padded table with all metal objects removed. A spine and anthropomorphic phantom were scanned each day of assessment to maintain quality assurance. DXA scans were performed and analyzed using standard Hologic analysis protocol. For statistical analysis, change in sub-total body fat mass was calculated as baseline measurements minus trial completion measurements; change in sub-total body lean mass was calculated as trial completion measurements minus baseline measurements.

### Statistics

Descriptive data are reported for variables of interest. Data were analyzed using SPSS Windows Version 18.0 (SPSS Inc., Chicago, IL). The associations between the variables were determined using the Pearson product moment coefficient of correlation.

A multiple linear regression model was constructed to determine the independent contribution of change in sub-total body fat mass (grams) and change in sub-total body lean mass (grams) on Stroop Test performance at trial completion. Baseline Stroop Test performance, age, baseline MMSE score, baseline FCI score, baseline GDS, and experimental group were statistically controlled by entering these six variables into the regression model first. These independent variables were determined from the results of the Pearson product moment coefficient of correlation analyses (i.e., baseline Stroop Test performance, age, baseline MMSE score, and baseline FCI score) or from assumed biological relevance (i.e., experimental group and GDS score). Both change in sub-total body fat mass and change in sub-total body lean mass were then entered into the regression model and only the variables that significantly improved the model were kept (i.e., significant Rsq change at *P*<0.05).

## Results

### Change in Variables of Interest

Age, GDS scores, MMSE scores, FCI scores, baseline Stroop performance, and average fat mass were similar across experimental groups. At the end of the 12-month trial, the 114 women who participated gained an average of 304.62 grams (0.67 pounds) of sub-total body fat mass and loss an average of 562.51 grams (1.24 pounds) of lean mass as measured by DXA. Stroop performance was improved by approximately four seconds. Based on normative data published from the Maastricht Aging study [45], a 5-second interval represents the difference in interference among women with average to high level of education between the mean ages of 65, 70, and 75 years. Table 1 reports values for variables of interest.

**Table 1:** Descriptive statistics for variables of interest.

	BAT (n = 36)		1× RT (n = 37)		2× RT (n = 41)		Total (N = 114)	
Variable	Mean	SD	Mean	SD	Mean	SD	Mean	SD
Age (years)	69.75	3.17	69.22	2.55	69.37	3.02	69.44	2.91
Height (cm)	161.96	6.66	160.68	6.24	162.28	6.32	161.67	6.39
Weight (kg)	68.50	10.92	68.63	12.52	70.84	14.68	69.39	12.84
BMI (kg/m^2^)	26.07	3.85	26.54	4.92	26.80	4.76	26.49	4.52
GDS (max. 15 pts)	0.61	2.06	0.11	0.66	0.95	2.50	0.57	1.95
MMSE (max. 30 pts)	28.81	1.19	28.84	1.19	28.49	1.57	28.70	1.34
FCI (max. 18 pts)	2.17	1.80	1.78	1.64	2.15	1.50	2.04	1.63
Baseline Stroop (sec)	43.89	15.43	47.09	27.01	46.37	16.61	45.82	20.14
Trial Completion Stroop (sec)	45.07	19.03	39.40	14.49	41.09	15.55	41.80	16.43
Δ in Stroop (sec)	−0.65	13.76	7.50	26.45	4.38	15.29	3.80	19.37
Baseline Sub-Total Fat Mass (grams)	24285.06	6755.59	25000.92	7989.44	26291.09	8605.68	25238.87	7835.59
Trial Completion Sub-Total Fat Mass (grams)	24677.41	7147.06	25057.10	8680.19	26742.87	8650.41	25543.49	8194.63
Δ in Sub-Total Fat Mass (grams)	392.34	2486.73	56.18	2329.93	451.79	2657.09	304.62	2484.85
Baseline Sub-Total Lean mass (grams)	37760.80	4472.11	37976.23	5143.97	38792.64	6808.14	38201.82	5589.02
Trial Completion Sub-Total Lean Mass (grams)	37198.28	4736.06	37507.19	5019.13	38272.34	6404.20	37684.83	5449.97
Δ Sub-Total Lean Mass (grams)	−562.51	1343.23	−469.04	1372.66	−520.31	1238.67	−517.00	1305.24

BAT = Balance and Tone; 1× RT = once-weekly resistance training; 2× RT = twice- weekly resistance training; BMI = weight in kilograms/height in square meters; Baseline Stroop and Trial completion Stroop performance = Stroop color words condition subtracted by Stroop coloured x's condition; Δ in Stroop = Stroop Baseline subtracted by Trial completion Stroop; Δ in Sub-total fat mass = Final fat mass subtracted by Baseline fat mass (a positive number represents an increase in fat mass and a negative number represents a decrease in fat mass); Δ in Sub-total lean mass = Final lean mass subtracted by Baseline lean mass (a positive number represents an increase in lean mass and a negative number represents a decrease in lean mass).

### Correlation Coefficients

Table 2 reports the correlation coefficients of those variables included in the final multi-variable regression model. Baseline Stroop performance, age, baseline MMSE, and baseline FCI were significantly associated with Stroop performance at trial completion (*P*<0.05). Change in sub-total body fat mass was significantly and negatively associated with the executive processes of selective attention and conflict resolution (*P*<0.05) – reduced fat mass was significantly associated with improved Stroop performance at trial completion. Experimental group, baseline GDS, and sub-total lean mass was not significantly associated with Stroop performance at trial completion (*P*>0.05).

**Table 2:** Multiple linear regression model assessing the contribution of fat and lean mass composition to trial completion Stroop test performance.

Independent Variables	r	R^2^	Adjusted R^2^	R^2^ Change	Unstandardized B (Standard Error)	Standardized β	*P* - Value
**Model 1**	0.591	0.356	0.320	0.356*			
Baseline Stroop	0.495*				0.362 (0.065)	0.444	0.000
Age	0.193*				0.464 (0.460)	0.082	0.315
MMSE	−0.334*				−2.482 (1.017)	−0.202	0.016
FCI	0.221*				1.808 (0.799)	0.180	0.026
GDS	0.071				0.091 (0.671)	0.011	0.893
Experimental Group	−0.096				−2.680 (1.564)	−0.134	0.090
**Model 2**	0.623	0.395	0.355	0.039*			
Baseline Stroop	0.495*				0.348 (0.064)	0.426	0.000
Age	0.193*				0.469 (0.448)	0.083	0.297
MMSE	−0.334*				−2.569 (0.991)	−0.209	0.011
FCI	0.221*				2.015 (0.782)	0.200	0.011
GDS	0.071				−0.179 (0.662)	−0.021	0.787
Experimental Group	−0.096				−2.675 (1.523)	−0.134	0.082
Δ Fat Mass	−0.213*				−0.001 (0.001)	−0.201	0.010
**Model 3**	0.630	0.403	0.358	0.008			
Baseline Stroop	0.495*				0.342 (0.064)	0.419	0.000
Age	0.193*				0.443 (0.447)	0.078	0.325
MMSE	−0.334*				−2.580 (0.989)	−0.210	0.010
FCI	0.221*				2.088 (0.783)	0.208	0.009
GDS	0.071				−0.273 (0.666)	−0.032	0.682
Experimental Group	−0.096				−2.638 (1.521)	−0.132	0.086
**Δ** Fat Mass	−0.213*				−0.001 (0.001)	−0.217	0.006
**Δ** Lean Mass	−0.089				−0.001 (0.001)	−0.091	0.241

* = significance at p<0.05.

Δ in Sub-total fat mass = Baseline fat mass subtracted by Final fat mass; Δ in Sub-total lean mass = Final lean mass subtracted by Baseline lean mass.

### Linear Regression Model

Baseline Stroop performance, age, baseline MMSE, baseline FCI, baseline GDS, and experimental group accounted for 35.6% of the variance in Stroop performance at trial completion. Adding change in sub-total body fat mass to the model resulted in a significant R-square change of 3.9% (*F* Change = 6.80, *P*<0.05). The total variance accounted by the final model was 39.5%. Change in lean mass was not a significant contribution to Stroop Test performance at trial completion (R-square change = 0.008, *F* Change = 1.390, *P*>0.05). Table 2 reports the results of our linear regression.

## Discussion

We found that reduced sub-total body fat mass was independently associated with better performance of selective attention and conflict resolution among community dwelling senior women. To our knowledge, this is the first study to examine the independent association of *change* in both sub-total body fat mass and sub-total body lean mass with executive functions after accounting for baseline cognitive performance, age, baseline global cognition, baseline number of comorbidities, baseline depression, and experimental group. Critically, we used radio-graphic measures of body composition which are more precise than measures of BMI or circumferences [46], [47].

Our finding of a significant independent association between reduced sub-total body fat mass and improved cognitive performance concurs and extends the results of previous studies [48], [49] examining the effects of obesity combined with hypertension on executive functions. These authors found a negative effect of obesity and hypertension on measures of memory [48] and executive functions [48], [49]. This negative association was attributed to brain pathologies caused by increased blood pressure facilitated by adipose tissue.

Our overall results are supported by biochemical studies demonstrating the negative effects of adipose metabolite secretion on brain health [14], [16], [18], [19], [20], [50], [51], [52]. Increased BMI is associated with increased plasma levels of inflammatory proteins such as C-reactive protein (CRP) and interleukin-6 (IL-6). Elevated levels of CRP and IL-6 reflect an enhanced inflammatory state and are associated with accelerated cognitive decline [51]. Specifically, Gorelick and colleagues [14] suggested that high levels of CRP and IL-6 resulted in nearly a 3-fold increase in vascular dementia risk. Critically, current evidence suggest that systemic inflammation is a key factor underlying the association between cardiovascular risk factors and neuronal damage [14].

Our findings extend the results of previous studies in the area of body composition and cognitive function in older adults [21], [22], [23], [24], [25], [32], [33]. Four previous studies assessed the association between fat mass and cognitive performance [21], [22], [24], [25], [32], [33], however, it remains unclear how *change* in fat mass may affect cognitive function.

To our knowledge, Han and colleagues [23] conducted the only study that included both baseline and follow-up measures of cognitive function and body fat mass, as measured by bioelectrical impedance, and found no significant associations between change in body fat mass and change in cognitive performance in adults aged 60–85 years. These contrasting results may be explained by differences in methodology – bioelectrical impedance analysis is a less accurate measure of body fat mass compared to DXA [53].

Furthermore, Han and colleges reported that changes in BMI, waist-to-hip ratio (WHR), and waist circumference (WC) were associated with changes in cognitive function. Obese men with increased BMI, WHR, and WC over time experienced a positive change in cognitive function. Normal weight women with reduced WC over time and obese women with reduced WHR over time experienced cognitive decline at follow-up. Thus, the relationship between adiposity and cognitive function may be dependent on the specific measure of body fat mass. As such, the effect of central adiposity versus total fat mass on cognitive performance warrants further investigation and standardization of the most valid and reliable method of measuring fat mass is necessary to understand the true relationship between adipose tissue and cognitive function in older adults.

Our study included the following limitations. Our study sample consisted exclusively of independent community-dwelling senior women who were without significant physical and cognitive impairments. Thus, the results of this study may not generalize to senior women with significant physical and/or cognitive impairments and we may have underestimated the contribution of change in body fat mass to selective attention and conflict resolution performance. Furthermore, the additional variance explained by sub-total body fat mass in the statistical model was only 3.9% (R-square change). Although this was statistically significant, it is unclear whether this overall effect results in a clinically important improvement. We note that the minimal mean change in sub-total body fat mass (i.e., 304.62 grams or ∼0.5 pounds) observed in this study may also underestimated the contribution of change in fat mass to selective attention and conflict resolution performance. Of note, the primary aim of the Brain POWER study intervention was to combat cognitive decline, not to change fat mass. As such, an intervention focused solely on affecting change in fat mass may show a larger effect.

Further studies are needed to provide a better understanding of the interplay between adiposity and cognitive function. Future studies may consider evaluating the effect of potential mediators that may lie in the causal pathway between adiposity and change in cognition. While prior studies have found that inflammatory factors are independently associated with cognitive decline [54], it is unclear how adipocytokines and metabolic variables affect cognitive function and whether they explain the effect of adiposity on cognitive function. Furthermore, visceral and subcutaneous fat tissue may differ in their production of various adipocytokines, such as adiponectin and leptin [55]. As such, it may be necessary to measure visceral and subcutaneous fat separately. In addition, other biochemical measures such as sex hormones may also help explain why men and women experience different outcomes in response to weight loss.

In conclusion, change in sub-total body fat mass – not change in lean mass – is independently associated with executive functions. This further emphasizes the potential value of targeted exercise training in combating cognitive decline [2], [56].

## References

[1] Alzheimer's Association (2012) 2012 Alzheimer's disease facts and figures. Alzheimers Dement 8: 131–168. 22404854 10.1016/j.jalz.2012.02.001

[2] Kramer AF, Erickson KI (2007) Capitalizing on cortical plasticity: influence of physical activity on cognition and brain function. Trends Cogn Sci 11: 342–348. 17629545 10.1016/j.tics.2007.06.009

[3] Kramer AF, Erickson KI, Colcombe SJ (2006) Exercise, cognition, and the aging brain. J Appl Physiol 101: 1237–1242. 16778001 10.1152/japplphysiol.00500.2006

[4] Liu-Ambrose T, Donaldson MG (2009) Exercise and cognition in older adults: is there a role for resistance training programmes? Br J Sports Med 43: 25–27. 19019904 10.1136/bjsm.2008.055616 PMC5298919

[5] Liu-Ambrose T, Donaldson MG, Ahamed Y, Graf P, Cook WL, et al (2008) Otago home-based strength and balance retraining improves executive functioning in older fallers: a randomized controlled trial. J Am Geriatr Soc 56: 1821–1830. 18795987 10.1111/j.1532-5415.2008.01931.x

[6] Liu-Ambrose T, Nagamatsu LS, Graf P, Beattie BL, Ashe MC, et al Resistance training and executive functions: a 12-month randomized controlled trial. Arch Intern Med 170: 170–178. 20101012 10.1001/archinternmed.2009.494 PMC3448565

[7] Cotman CW, Berchtold NC (2002) Exercise: a behavioral intervention to enhance brain health and plasticity. Trends Neurosci 25: 295–301. 12086747 10.1016/s0166-2236(02)02143-4

[8] Lindvall O, Kokaia Z, Bengzon J, ElmeÂ'r E, Kokaia M (1994) Neurotrophins and brain insults. Trends Neurosci 17: 490–496. 7531892 10.1016/0166-2236(94)90139-2

[9] Hu G, Barengo NC, Tuomilehto J, Lakka TA, Nissinen A, et al (2004) Relationship of physical activity and body mass index to the risk of hypertension: a prospective study in Finland. Hypertension 43: 25–30. 14656958 10.1161/01.HYP.0000107400.72456.19

[10] Oguma Y, Shinoda-Tagawa T (2004) Physical activity decreases cardiovascular disease risk in women: review and meta-analysis. Am J Prev Med 26: 407–418. 15165657 10.1016/j.amepre.2004.02.007

[11] Lee CD, Folsom AR, Blair SN (2003) Physical activity and stroke risk: a meta-analysis. Stroke 34: 2475–2481. 14500932 10.1161/01.STR.0000091843.02517.9D

[12] Poirier P, Giles TD, Bray GA, Hong Y, Stern JS, et al (2006) Obesity and cardiovascular disease: pathophysiology, evaluation, and effect of weight loss: an update of the 1997 American Heart Association Scientific Statement on Obesity and Heart Disease from the Obesity Committee of the Council on Nutrition, Physical Activity, and Metabolism. Circulation 113: 898–918. 16380542 10.1161/CIRCULATIONAHA.106.171016

[13] Gustafson D (2008) A life course of adiposity and dementia. Eur J Pharmacol 585: 163–175. 18423446 10.1016/j.ejphar.2008.01.052

[14] Gorelick PB, Scuteri A, Black SE, DeCarli C, Greenberg SM, et al (2011) Vascular Contributions to Cognitive Impairment and Dementia: A Statement for Healthcare Professionals From the American Heart Association/American Stroke Association. Stroke 42: 2672–2713. 21778438 10.1161/STR.0b013e3182299496 PMC3778669

[15] Jellinger KA (2008) The Pathology of “Vascular Dementia”: A Critical Update. J Alzheimers Dis 14: 107–123. 18525132 10.3233/jad-2008-14110

[16] Grammas P, Ovase R (2002) Cerebrovascular transforming growth factor-beta contributes to inflammation in the Alzheimer's disease brain. Am J Pathol 160: 1583–1587. 12000710 10.1016/s0002-9440(10)61105-4 PMC1850858

[17] McCusker SM, Curran MD, Dynan KB, McCullagh CD, Urquhart DD, et al (2001) Association between polymorphism in regulatory region of gene encoding tumour necrosis factor alpha and risk of Alzheimer's disease and vascular dementia: a case-control study. Lancet 357: 436. 11273064 10.1016/s0140-6736(00)04008-3

[18] Savaskan E, Hock C, Olivieri G, Bruttel S, Rosenberg C, et al (2001) Cortical alterations of angiotensin converting enzyme, angiotensin II and AT1 receptor in Alzheimer's dementia. Neurobiol Aging 22: 541–546. 11445253 10.1016/s0197-4580(00)00259-1

[19] Luterman JD, Haroutunian V, Yemul S, Ho L, Purohit D, et al (2000) Cytokine gene expression as a function of the clinical progression of Alzheimer disease dementia. Arch Neurol 57: 1153–1160. 10927795 10.1001/archneur.57.8.1153

[20] Craft S (2009) The role of metabolic disorders in Alzheimer disease and vascular dementia: two roads converged. Arch Neurol 66: 300–305. 19273747 10.1001/archneurol.2009.27 PMC2717716

[21] Dore GA, Elias MF, Robbins MA, Budge MM, Elias PK (2008) Relation between central adiposity and cognitive function in the Maine-Syracuse study: attenuation by physical activity. Ann Behav Med 35: 341–350. 18584267 10.1007/s12160-008-9038-7

[22] Yoon DH, Choi SH, Yu JH, Ha JH, Ryu SH, et al (2012) The relationship between visceral adiposity and cognitive performance in older adults. Age Ageing 41: 456–461. 22440588 10.1093/ageing/afs018

[23] Han C, Jo SA, Seo JA, Kim BG, Kim NH, et al (2009) Adiposity parameters and cognitive function in the elderly: application of “Jolly Fat” hypothesis to cognition. Arch Gerontol Geriatr 49: e133–138. 19108905 10.1016/j.archger.2008.11.005

[24] Kanaya AM, Lindquist K, Harris TB, Launer L, Rosano C, et al (2009) Total and regional adiposity and cognitive change in older adults: The Health, Aging and Body Composition (ABC) Study. Arch Neurol 66: 329–335. 19273751 10.1001/archneurol.2008.570 PMC2739693

[25] Driscoll I, Espeland MA, Wassertheil-Smoller S, Gaussoin SA, Ding J, et al (2011) Weight change and cognitive function: findings from the Women's Health Initiative Study of Cognitive Aging. Obesity (Silver Spring) 19: 1595–1600. 21394095 10.1038/oby.2011.23 PMC3175491

[26] Fitzpatrick AL, Kuller LH, Lopez OL, Diehr P, O'Meara ES, et al (2009) Midlife and late-life obesity and the risk of dementia: cardiovascular health study. Arch Neurol 66: 336–342. 19273752 10.1001/archneurol.2008.582 PMC3513375

[27] Hughes TF, Borenstein AR, Schofield E, Wu Y, Larson EB (2009) Association between late-life body mass index and dementia: The Kame Project. Neurology 72: 1741–1746. 19451529 10.1212/WNL.0b013e3181a60a58 PMC2683740

[28] Kivipelto M, Ngandu T, Fratiglioni L, Viitanen M, Kareholt I, et al (2005) Obesity and vascular risk factors at midlife and the risk of dementia and Alzheimer disease. Arch Neurol 62: 1556–1560. 16216938 10.1001/archneur.62.10.1556

[29] Whitmer RA, Gunderson EP, Barrett-Connor E, Quesenberry CPJ, Yaffe K (2005) Obesity in middle age and future risk of dementia: a 27 year longitudinal population based study. BMJ 330: 1360. 15863436 10.1136/bmj.38446.466238.E0 PMC558283

[30] Kyle UG, Genton L, Hans D, Karsegard L, Slosman DO, et al (2001) Age-related differences in fat-free mass, skeletal muscle, body cell mass and fat mass between 18 and 94 years. Eur J Clin Nutr 55: 663–672. 11477465 10.1038/sj.ejcn.1601198

[31] Burkhauser RV, Cawley J (2008) Beyond BMI: the value of more accurate measures of fatness and obesity in social science research. J Health Econ 27: 519–529. 18166236 10.1016/j.jhealeco.2007.05.005

[32] Bagger YZ, Tanko LB, Alexandersen P, Qin G, Christiansen C (2004) The implications of body fat mass and fat distribution for cognitive function in elderly women. Obes Res 12: 1519–1526. 15483217 10.1038/oby.2004.189

[33] Kuo HK, Jones RN, Milberg WP, Tennstedt S, Talbot L, et al (2006) Cognitive function in normal-weight, overweight, and obese older adults: an analysis of the Advanced Cognitive Training for Independent and Vital Elderly cohort. J Am Geriatr Soc 54: 97–103. 16420204 10.1111/j.1532-5415.2005.00522.x PMC2834231

[34] Jacqmin-Gadda H, Fabrigoule C, Commenges D, Dartigues JF (1997) A 5-year longitudinal study of the Mini-Mental State Examination in normal aging. Am J Epidemiol 145: 498–506. 9063339 10.1093/oxfordjournals.aje.a009137

[35] Royall DR, Palmer R, Chiodo LK, Polk MJ (2004) Declining executive control in normal aging predicts change in functional status: The Freedom House Study. J Am Geriatr Soc 52: 346–352. 14962147 10.1111/j.1532-5415.2004.52104.x

[36] Baddeley A (1996) Exploring the central executive. Q J Exp Psychol (Hove): Section A 49: 5–28.

[37] Spreen O, Strauss E (1998) A compendium of neurological tests: administration, norms, and commentary. New York: Oxford University Press.

[38] Liu-Ambrose T, Pang MYC, Eng JJ (2007) Executive function is independently associated with performances of balance and mobility in community-dwelling older adults after mild stroke: implications for falls prevention. Cerebrovasc Dis (Basel, Switzerland) 23: 203–210. 10.1159/000097642 PMC4492718 17143004

[39] Jefferson AL, Paul RH, Ozonoff A, Cohen RA (2006) Evaluating elements of executive functioning as predictors of instrumental activities of daily living (IADLs). Arch Clin Neuropsychol 21: 311–320. 16814980 10.1016/j.acn.2006.03.007 PMC2746400

[40] Tabert MH, Manly JJ, Liu X, Pelton GH, Rosenblum S, et al (2006) Neuropsychological prediction of conversion to Alzheimer disease in patients with mild cognitive impairment. Arch Gen Psychiatry 63: 916–924. 16894068 10.1001/archpsyc.63.8.916

[41] Liu-Ambrose T, Nagamatsu LS, Graf P, Beattie BL, Ashe MC, et al (2010) Resistance training and executive functions: a 12-month randomized controlled trial. Arch Intern Med 170: 170–178. 20101012 10.1001/archinternmed.2009.494 PMC3448565

[42] Folstein MF, Folstein SE, McHugh PR (1975) “Mini-mental state”. A practical method for grading the cognitive state of patients for the clinician. J Psychiatr Res 12: 189–198. 1202204 10.1016/0022-3956(75)90026-6

[43] Yesavage JA (1988) Geriatric Depression Scale. Psychopharmacol Bull 24: 709–711. 3249773

[44] Groll DL, To T, Bombardier C, Wright JG (2005) The development of a comorbidity index with physical function as the outcome. J Clin Epidemiol 58: 595–602. 15878473 10.1016/j.jclinepi.2004.10.018

[45] Van der Elst W, Van Boxtel MP, Van Breukelen GJ, Jolles J (2006) The Stroop color-word test: influence of age, sex, and education; and normative data for a large sample across the adult age range. Assessment 13: 62–79. 16443719 10.1177/1073191105283427

[46] Haarbo J, Gotfredsen A, Hassager C, Christiansen C (1991) Validation of body composition by dual energy X-ray absorptiometry (DEXA). Clin Physiol 11: 331–341. 1914437 10.1111/j.1475-097x.1991.tb00662.x

[47] Romero-Corral A, Somers VK, Sierra-Johnson J, Thomas RJ, Collazo-Clavell ML, et al (2008) Accuracy of body mass index in diagnosing obesity in the adult general population. Int J Obes (Lond) 32: 959–966. 18283284 10.1038/ijo.2008.11 PMC2877506

[48] Elias MF, Elias PK, Sullivan LM, Wolf PA, D'Agostino RB (2003) Lower cognitive function in the presence of obesity and hypertension: the Framingham Heart Study. Int J Obes Relat Metab Disord 27: 260–268. 12587008 10.1038/sj.ijo.802225

[49] Waldstein SR, Katzel LI (2006) Interactive relations of central versus total obesity and blood pressure to cognitive function. Int J Obes (Lond) 30: 201–207. 16231030 10.1038/sj.ijo.0803114

[50] Katagiri H, Yamada T, Oka Y (2007) Adiposity and cardiovascular disorders: disturbance of the regulatory system consisting of humoral and neuronal signals. Circ Res 101: 27–39. 17615379 10.1161/CIRCRESAHA.107.151621

[51] Yaffe K, Lindquist K, Penninx BW, Simonsick EM, Pahor M, et al (2003) Inflammatory markers and cognition in well-functioning African-American and white elders. Neurology 61: 76–80. 12847160 10.1212/01.wnl.0000073620.42047.d7

[52] Ziccardi P, Nappo F, Giugliano G, Esposito K, Marfella R, et al (2002) Reduction of inflammatory cytokine concentrations and improvement of endothelial functions in obese women after weight loss over one year. Circulation 105: 804–809. 11854119 10.1161/hc0702.104279

[53] Svendsen OL, Haarbo J, Heitmann BL, Gotfredsen A, Christiansen C (1991) Measurement of body fat in elderly subjects by dual-energy x-ray absorptiometry, bioelectrical impedance, and anthropometry. Am J Clin Nutr 53: 1117–1123. 2021122 10.1093/ajcn/53.5.1117

[54] Yaffe K, Kanaya A, Lindquist K, Simonsick EM, Harris T, et al (2004) The metabolic syndrome, inflammation, and risk of cognitive decline. JAMA 292: 2237–2242. 15536110 10.1001/jama.292.18.2237

[55] Park KG, Park KS, Kim MJ, Kim HS, Suh YS, et al (2004) Relationship between serum adiponectin and leptin concentrations and body fat distribution. Diabetes Res Clin Pract 63: 135–142. 14739054 10.1016/j.diabres.2003.09.010

[56] Colcombe SJ, Kramer AF, Erickson KI, Scalf P, McAuley E, et al (2004) Cardiovascular fitness, cortical plasticity, and aging. Proc Natl Acad Sci U S A 101: 3316–3321. 14978288 10.1073/pnas.0400266101 PMC373255

